# HSF-1/miR-145-5p transcriptional axis enhances hyperthermic intraperitoneal chemotherapy efficacy on peritoneal ovarian carcinosis

**DOI:** 10.1038/s41419-023-06064-9

**Published:** 2023-08-19

**Authors:** Silvia Di Agostino, Valeria Canu, Sara Donzelli, Claudio Pulito, Andrea Sacconi, Federica Ganci, Fabio Valenti, Frauke Goeman, Stefano Scalera, Francesca Rollo, Anna Bagnato, Maria Grazia Diodoro, Enrico Vizza, Mariantonia Carosi, Beatrice Rufini, Orietta Federici, Manuel Giofrè, Fabio Carboni, Paola Muti, Gennaro Ciliberto, Sabrina Strano, Mario Valle, Giovanni Blandino

**Affiliations:** 1 Department of Health Sciences, Magna Græcia University of Catanzaro, 88100 Catanzaro, Italy; 2 Translational Oncology Research Unit, Department of Research, Diagnosis and Innovative Technologies, IRCCS Regina Elena National Cancer Institute, Rome, Italy; 3 Clinical Trial Center, Biostatistics and Bioinformatics Unit, IRCCS Regina Elena National Cancer Institute, Rome, Italy; 4 SAFU Unit, Department of Research, Diagnosis and Innovative Technologies, IRCCS Regina Elena National Cancer Institute, Rome, Italy; 5 Department of Pathology, IRCCS Regina Elena National Cancer Institute, Rome, Italy; 6 Preclinical Models and New Therapeutic Agents Unit, IRCCS-Regina Elena National Cancer Institute, Rome, Italy; 7 Gynecologic Oncology Unit, Department of Experimental Clinical Oncology, IRCCS Regina Elena National Cancer Institute, Rome, Italy; 8 Department of Digestive Surgery, IRCCS Regina Elena National Cancer Institute, Rome, Italy; 9 Department of Biomedical, Surgical and Dental Sciences, University of Milan, Milan, Italy; 10 Scientific Direction, IRCCS Regina Elena National Cancer Institute, 00144 Rome, Italy

**Keywords:** Cancer, Translational research

## Abstract

Hyperthermic intraperitoneal administration of chemotherapy (HIPEC) increases local drug concentrations and reduces systemic side effects associated with prolonged adjuvant intraperitoneal exposure in patients affected by either peritoneal malignancies or metastatic diseases originating from gastric, colon, kidney, and ovarian primary tumors. Mechanistically, the anticancer effects of HIPEC have been poorly explored. Herein we documented that HIPEC treatment promoted miR-145-5p expression paired with a significant downregulation of its oncogenic target genes c-MYC, EGFR, OCT4, and MUC1 in a pilot cohort of patients with ovarian peritoneal metastatic lesions. RNA sequencing analyses of ovarian peritoneal metastatic nodules from HIPEC treated patients unveils HSF-1 as a transcriptional regulator factor of miR-145-5p expression. Notably, either depletion of HSF-1 expression or chemical inhibition of its transcriptional activity impaired miR-145-5p tumor suppressor activity and the response to cisplatin in ovarian cancer cell lines incubated at 42 °C. In aggregate, our findings highlight a novel transcriptional network involving HSF-1, miR145-5p, MYC, EGFR, MUC1, and OCT4 whose proper activity contributes to HIPEC anticancer efficacy in the treatment of ovarian metastatic peritoneal lesions.

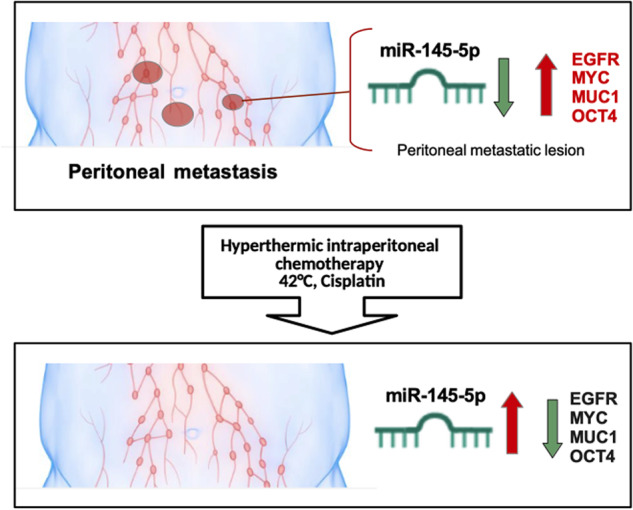

## Introduction

High-grade serous ovarian cancer (HGSOC) and ovarian carcinosarcoma (OCS) are the most aggressive types of ovarian cancer that occurs in women with and without a genetic predisposition [1, 2]. HGSOC is the most common type of ovarian cancer, accounting for approximately 75% of epithelial ovarian cancers [2].

A recent analysis by COSMIC database (COSMIC v92, Catalog of Somatic Mutation in Cancer; https://cancer.sanger.ac.uk/cosmic/) identified *TP53* (98%), *KRAS* (6%), *NF1* (6%), *BRCA1/2* (5%), and *ATR* (5%) as the six genes frequently mutated in HGSOC contributing to high genomic instability, intraperitoneal spreading (carcinomatosis) and distant metastases [3, 4]. The majority of OC metastasize to adjacent sites along the peritoneum throughout the pelvic and abdominal cavity [3–5]. Although 75% of patients with advanced disease respond to chemotherapy initially (cisplatin-containing), many of them relapse within 2 years after completing treatment acquiring the resistance to chemotherapy [5]. The lack of early diagnosis together with the high incidence of tumor relapse and acquired resistance to chemotherapy contribute to the difficulties in developing precise intervention and therapy for the patients.

Hyperthermic intraperitoneal chemotherapy (HIPEC) is a highly concentrated, heated chemotherapy treatment (42 °C) that is directly delivered into the abdomen during surgery [6–8]. Unlike systemic chemotherapy delivery, which circulates around the body through the bloodstream, HIPEC delivers chemotherapy directly to the metastatic lesion allowing the use of higher doses of chemotherapy treatment [6]. Heating the chemotherapy solution may also improve the absorption of the drugs by tumors and destroy cancer cells that remain in the abdomen after surgery. HIPEC showed an experimental increase in the survival of most patients without negatively affecting quality of life [6–9]. However, the molecular and biological mechanisms that determine the improvement of patients’ health treated with HIPEC compared to the traditional chemotherapeutic protocol are still unknown.

MicroRNAs (miRNAs) are short non-coding RNA molecules (19–24 nucleotides long) which due to their presence in large quantities in tissues and fluids, good stability and their role as gene expression regulators, are emerging as potential biomarkers in different types of cancer [10, 11]. Aberrant miRNA expression showed diagnostic, prognostic, and therapeutic implications [12–14]. MiRNAs are detected in human body fluids suggesting that they can perform functions on cells of distant organs, mediating both short- and long-range cell-to-cell communication [15, 16].

Hsa-miR-145 is recognized by many studies to be a potent tumor suppressor miRNA in various types of cancer, including ovarian cancer, by inhibiting tumor cell proliferation, invasion, and metastasis and potentiating the anticancer effects of chemotherapy drugs [17–23]. It has been reported that low miR-145-5p expression was associated with a metastatic phenotype thereby suggesting that miR-145-5p could be considered a metastamiR, which is an miR associated with metastatic pathways [22]. We have previously shown that miR-145-5p expression was significantly reduced in brain metastasis derived from lung cancer and melanoma when compared to matched primary cancer tissues [22].

MiR-145-5p exerts its biological effects through the down-regulation of its downstream target genes, some of which have already been validated such as octamer-binding transcription factor 4 (*OCT4*), SRY-box 9 (*SOX9*), *c-MYC*, *YES*, epidermal growth factor receptor (*EGFR*), insulin-like growth factor (*IGF*), mucin 1 (*MUC1*), signal transducer and activator of transcription 1 (*STAT1*), tumor necrosis factor super family member 10 (*TNFSF10*) and many others [19, 22–26]. Recent studies reported that miR-145 is an emerging non-invasive tool for monitoring invasion and migration of diverse tumors, indicating that it may serve as a non-invasive biomarker for clinical application [27, 28]. Importantly, low levels of miR-145 in the cancer tissues and in the serum are significantly associated with relapse and poor prognosis in ovarian cancer patients [23, 29].

Here, we aimed to study the contribution of miR-145-5p tumor suppressor activities to HIPEC treatment of ovarian peritoneal metastases. In particular, we found that HIPEC treatment of ovarian peritoneal metastatic lesions restored miR-145-5p expression leading to the down-regulation of its validated target genes MYC, EGFR, MUC1, and OCT4 when compared to untreated-matched tumoral tissues. RNA sequencing analysis of the peritoneal metastatic nodules treated with HIPEC revealed HSF-1 as a transcriptional regulator of miR-145-5p expression. Indeed, we found that HSF-1 directly and specifically binds to its DNA consensus sequences on the promoter of miR-145-5p in an in vitro OC cell model of HIPEC. Furthermore, we observed that the restored expression of miR-145-5p by incubating at 42 °C or by treating ovarian cancer cells with DNA methyltransferase 5-Aza-deoxycytidine both significantly reduced cell viability.

## Results

### Molecular characterization of ovarian peritoneal metastatic lesions and evaluation of miR-145-5p and its target gene expression

**Fig. 1:**
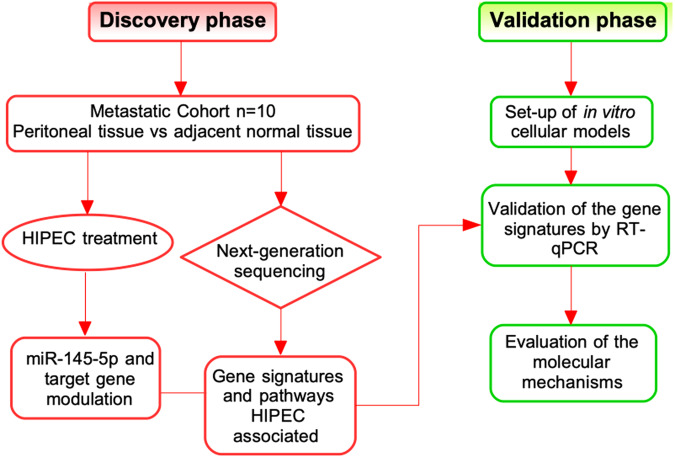
Flowchart of the study design. The figure illustrates the flowchart of discovery (red) and validation (green) phase of this study. The details are described in the text.

Ten HIPEC eligible patients affected by ovarian peritoneal carcinomatosis were included in the pilot study. A flowchart of the entire study design is illustrated in the Fig. 1.

First, we collected metastatic peritoneal sample and peritoneal tissue free of any metastatic histological sign from each patient. Second, we collected metastatic nodule pieces at 0, 15, 30, 60, and 90 min after HIPEC therapeutic perfusion. Tissues at time point 0 min represented HIPEC-untreated metastatic lesions. Clinical data of the analyzed patients including age at surgery, grade and stage, FIGO classification, disease course, previous therapies and clinical outcomes were summarized in Supplementary Table 1.

To molecularly characterize the peritoneal carcinomatosis collected, NGS sequencing for *TP53* and *BRCA1/2* genes was performed. As reported in Supplementary Table 2, 9 out of 10 patients (90%) carried *TP53* gene mutations while only 5 patients (50%) exhibited *BRCA1/BRCA2* gene mutations. Interestingly, all of the identified *TP53* missense mutations occur in the DNA binding domain of p53 proteins (Supplementary Fig. S1A). Hyperplastic ovarian tissue was considered as a negative control tissue for the analysis. Ovarian hyperplasia is characterized by a proliferation of stroma of the ovary, it is a pre-cancerous stage that did not reveal any specific mutations of *TP53* and *BRCA1/2* genes. DNA sequencing results were in line with the data reported in the literature about the occurrence of these mutations in the primary HGSOC and its metastasis [30–32].

Immunohistochemistry analysis (IHC) revealed intense nuclear p53 protein staining for accumulated mutant p53 proteins and no signal for BRCA1 protein staining in metastatic tissues [5, 30–32] (Fig. 1A and Supplementary Fig. S1B). Unlike peritoneal metastatic tissues, no p53 staining, for short half-life wt-p53 protein and detectable BRCA1 protein were observed in hyperplastic tissue (Fig. 1A and Supplementary Fig. S1B). These findings paired with those extensively reported in literature agreed that OC carrying either somatic or germline *BRCA1/BRCA2* mutations are uniformly accompanied by p53 dysfunction [30, 31].

**Fig. 2:**
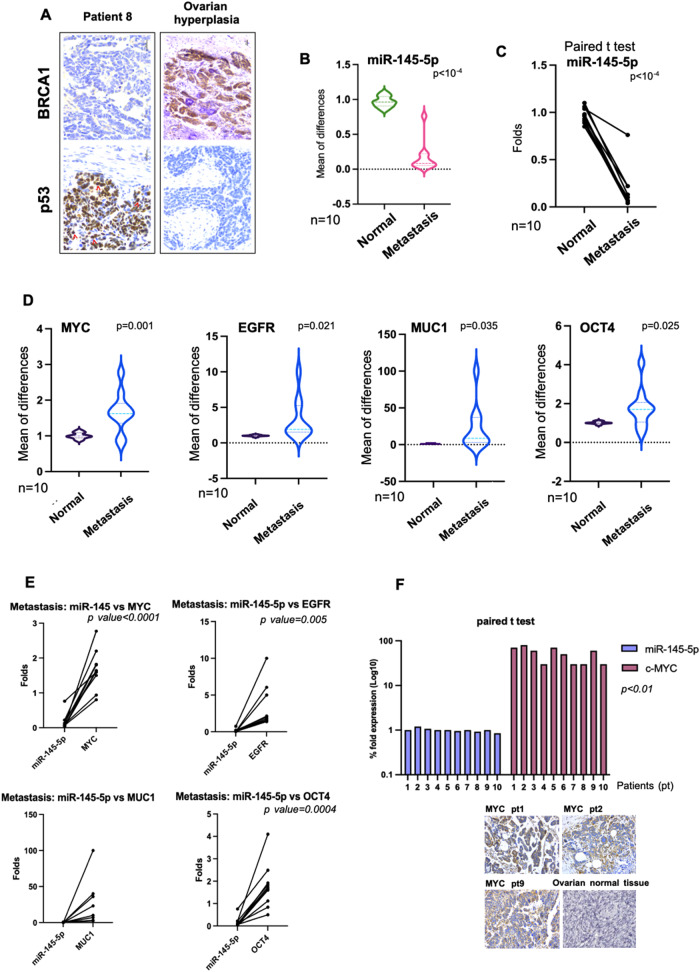
Molecular characterization of ovarian peritoneal metastatic lesions and evaluation of miR-145-5p and its target gene expression. **A** Staining by H&E and IHC for the indicated proteins of peritoneal metastasis from a representative tissue of IRE patients. Tissue from ovarian dysplasia was hybridized with the same antibodies as positive control. Original magnification, 40x. Red arrows indicate examples of cells with complete nuclear staining of p53. **B** Quantitative real-time PCR analysis showing miR-145-5p expression in metastatic ovarian cancer samples (Metastasis) compared with normal adjacent tissue samples (Normal). The expression levels are represented by violin plots. All expression values were normalized to small nuclear RNU6. **C** Paired *t*-test analysis (two tailed) between the expression of miR-145-5p in metastatic ovarian cancer samples paired with normal adjacent tissues of patients. **D** Quantitative real-time PCR analysis of *MYC, EGFR, MUC1* and *OCT4* expression in metastatic ovarian cancer samples (Metastasis) compared with normal adjacent tissue samples (Normal). The expression levels are represented by violin plots. *P* values are indicated on the graph. **E** Paired *t*-test analysis (two tailed) between the expression of miR-145-5p versus the expression of its target genes in each patient, in the metastatic samples. Significantly different *p* < 0.05. **F** The graph showed paired *t*-test analysis (two tailed) between expression of miR-145-5p (RNA) and MYC protein in peritoneal metastasis samples. Correlation coefficient (R) was −0.693. Staining by H&E and IHC for MYC expression from representative tissues of IRE patients (pt). As control, we reported normal ovarian tissue staining by The Human Protein Atlas free database, where MYC expression was negative by using three different antibodies. https://www.proteinatlas.org/ENSG00000136997-MYC/tissue/ovary. Original magnification of all images was 40x.

It has been previously reported that reduced miR-145-5p expression was associated with metastatic process [22, 33]. This prompted us to assess the expression of miR-145-5p in peritoneal metastatic lesions and monitor its expression upon HIPEC treatment. Firstly, we analyzed the expression of miR-145-5p comparing non-tumoral peritoneal tissues with the matched metastatic peritoneal lesion of all ten patients. As shown in Fig. 2B, C, the expression of miR-145-5p was significantly reduced in the metastatic tissues. Secondly, we assessed the expression of four well-validated miR-145-5p mRNA targets, such as *MYC, EGFR, MUC1,* and *OCT4*, in the same tissues [22–26]. All four genes and their derived proteins are closely involved in the metastatic process [22, 34]. We found that all gene transcript expressions were significantly upregulated (Fig. 2D). Furthermore, the results were consolidated by the paired *t*-test analysis comparing the expression of miR-145-5p with that of its target genes in metastatic tissues (Fig. 2E). Notably, miR-145-5p and MYC protein expression, the latter obtained by IHC staining, was significantly anti-correlated in the metastatic tissues (R = −0.693; *p* < 0.01) (Fig. 2F).

Altogether, our findings documented the aberrant and anti-correlated expression of miR-145-5p and the *MYC, EGFR, MUC1*, and *OCT4* target genes in ovarian peritoneal metastatic lesions.

### HIPEC treatment restores the expression of miR-145-5p and modulates HIPEC-specific pathways in peritoneal metastatic lesions

**Fig. 3:**
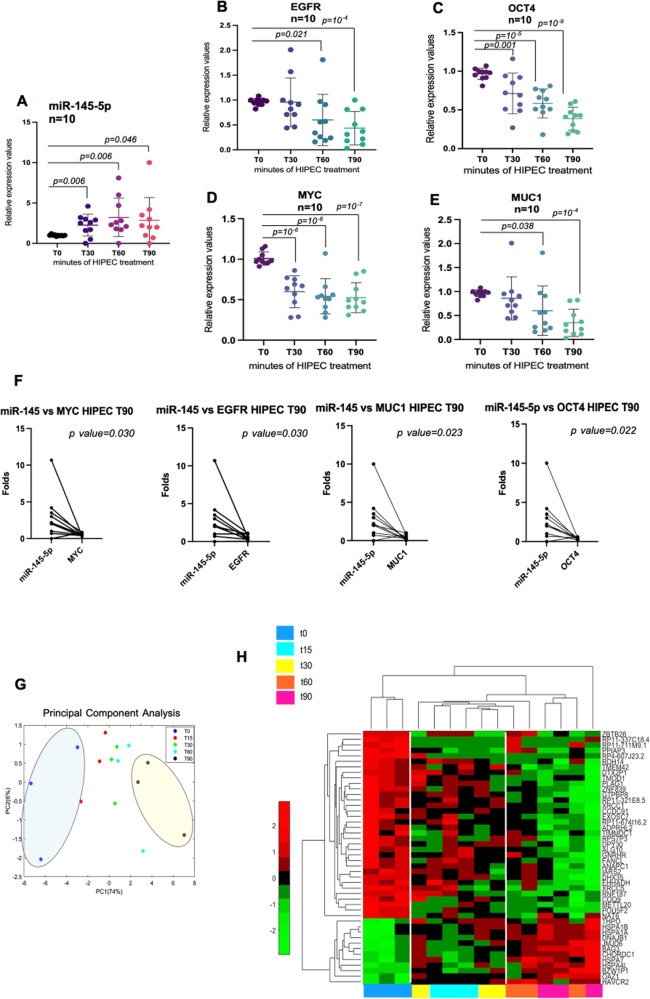
HIPEC treatment restores the expression of miR-145-5p and modulates HIPEC-specific pathways in peritoneal metastatic lesions. **A**–**E** Scatter diagram graphs showing the expression of miR-145-5p, EGFR, OCT4, MYC, and MUC1, respectively, from the metastatic tissue samples during the time of the HIPEC treatment. *p* values are indicated on the graph. **F** Paired *t*-test analysis (two tailed) between the expression of miR-145-5p versus the HIPEC lesion samples at T90. Significantly different *p* < 0.05. **G** PCA plot shows the 3 patients considered in the RNA sequencing experiment distinguishing them in the various treatment times. In particular, the image highlighted the patients at T0 (blue oval) and T90 (yellow oval) time of CDDP perfusion. **H** The heat-map of differential expressed genes by RNA-seq between the indicated time of HIPEC treatment. The colors of the time of HIPEC treatment are indicated in the legend of the image.

We aimed to assess whether HIPEC treatment affected the anti-correlated expression of miR-145-5p and its mRNA target genes. To this end, total RNA was extracted from peritoneal ovarian metastatic nodule at different times (T30, T60, T90) upon HIPEC treatment and analysed in comparison with T0 tissue. In particular, we found that the expression of miR-145-5p was significantly up-regulated at all the analysed times upon HIPEC treatment when compared with T0 metastatic lesion (Fig. 3A). We subsequently evaluated the expression of *MYC, EGFR, MUC1,* and *OCT4* genes and found that their expression was significantly downregulated at the different time points upon HIPEC treatment (Fig. 3B–E). In particular, while the expression of *OCT4* and *MYC* was already significantly downregulated at T30, T60, and T90, that of *EGFR,* and *MUC1* was reduced at T60 and T90 upon HIPEC treatment (Fig. 3B–E). Notably, the data was further strengthened by the paired *t-*test analysis between the expression of miR-145-5p versus the expression of its target genes in each patient at least at T60 and T90 that resulted significant (Fig. 3F).

Overall, the reported findings strongly indicated that HIPEC treatment reverts the aberrant and anti-correlated expression of miR-145-5p and *MYC, EGFR, MUC1*, and *OCT4* genes in peritoneal ovarian metastatic tissue.

To further decipher the impact of HIPEC on global gene expression of the peritoneal metastatic nodules, we performed RNA-seq analysis of three HIPEC eligible patients (T0, T15, T30, T60, T90). Principle Component Analysis (PCA) revealed that patient treatment at different time points could be visualized on PC1 (74%) and PC2 (6%) respectively (Fig. 3G). Remarkably, despite the small number of analysed patients, differential gene expression significantly grouped patient metastatic nodules by HIPEC treatment and time of treatment. Untreated metastatic lesions (T0) were noticeably distinguished from T90 on PC1 while T15, T30, and T60 clustered with intermediate gene expression profile compared to those at T0 and T90, respectively (Fig. 3G). By performing the Unsupervised Hierarchical Analysis, we found 46 differential expressed genes (DEGs) that were significantly modulated between T0 and T90 (Fig. 3H and Supplementary Table 3). Altogether, our findings imply that HIPEC treatment instigated a fast and selective modulation of gene expression profile when compared to untreated metastatic lesions, and further analyses on these identified genes will be described later in this study.

### Ectopic expression of miR-145-5p acts as tumor suppressor in ovarian cancer cell lines

**Fig. 4:**
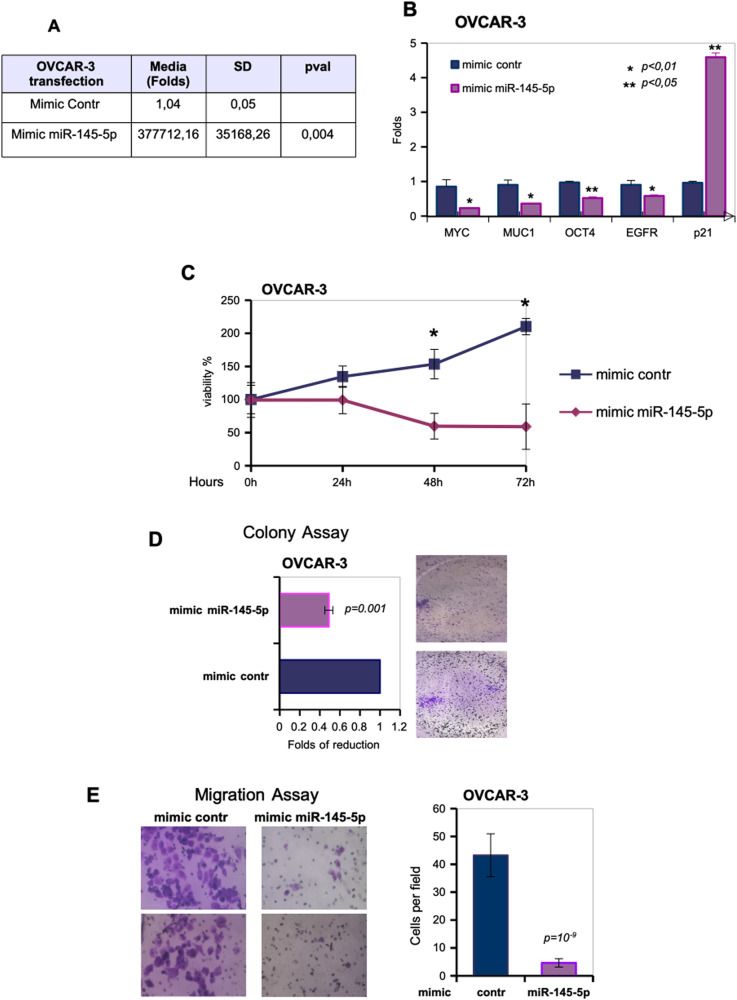
Ectopic expression of miR-145-5p acts as tumor suppressor in ovarian cancer cell lines. **A** OVCAR-3 cell line was transfected with oligonucleotides overexpressing miR-145-5p (mimic miR-145-5p) and mimic control as a negative sample. RT-qPCR by TaqMan assay was performed to test the overexpression. In the figure, the relative fold induction of miR-145-5p expression over the negative control sample are reported. TaqMan assay for RNU48 amplification was used to normalize the expression values. **B** *MYC, MUC1, OCT4, EGFR*, and *p21* expression were analyzed by RT-qPCR in cDNA derived from OVCAR-3 cells described in (**A**). *p* values of the real time PCRs were calculated with two-tailed Student’s *t*-test. Statistically significant results are indicated in the figures. **C** Viabilty of OVCAR-3 cells transfected with mimic miR-145-5p or mimic control was tested by MTT assay at the indicated time points. Values are mean ± SD obtained from 2 separate experiments in quintuplicate. *p* values were calculated with two-tailed *t*-test. Significant *p* values are indicated as *p < 0.01 versus the control sample. **D** Representative images and quantification of the transwell migration assay by Boyden chamber in OVCAR-3 cells. Magnification, 40x. Data in the graph represent the mean ± SD from three biological replicates of the transwell migration assay, each point repeated in technical quadruplicates. *p* values were calculated with two-tailed *t*-test. **E** Colony formation assay was performed in OVCAR-3 cells transfected with mimic control and mimic-miR-145-5p. The data was obtained by analysing the colonies with ImageJ software. *p* values were calculated with two-tailed *t*-test.

To explore miR-145-5p tumor suppressor activities preclinically, we evaluated the effects provoked by the ectopic expression of mimic miR-145-5p compared to control mimic in OVCAR-3 and ES2 ovarian cancer cell lines (Fig. 4A and Fig. S2A). Firstly, mimic-miR-145-5p transduction significantly reduced the expression levels of *MYC, EGFR, MUC1,* and *OCT4* target genes in OVCAR-3 and ES2 cell lines when compared to control mimic (Fig. 4B and Fig. S2A). Unlike miR-145-5p target genes, *p21WAF1* transcript was upregulated as it frequently occurs upon elevated expression of tumor suppressor genes and concomitantly, we observed the induction of the expression of some apoptotic targets such as CHOP, BAX, and NOXA (Fig. 4B and Fig. S2A, B). Indeed, ectopic transduction of miR-145-5p determined a significant reduction of OVCAR-3 cell proliferation (Fig.4C and Fig. S2C) pairing with a significant reduction of colony formation of both OVCAR-3 and ES2 cell lines (Fig. 4D and Fig. S2D). Furthermore, miR-145-5p overexpression strongly impaired migration of ovarian cancer cells when compared to cells transduced with control mimic (Fig. 4E and Fig. S2E).

In conclusion, the reported findings clearly showed that miR-145-5p exerts tumor suppressor effects in ovarian cancer cell lines.

### Enrichment of mature miR-145-5p occurs in ovarian cancer cells upon HIPEC-mimicking treatment

**Fig. 5:**
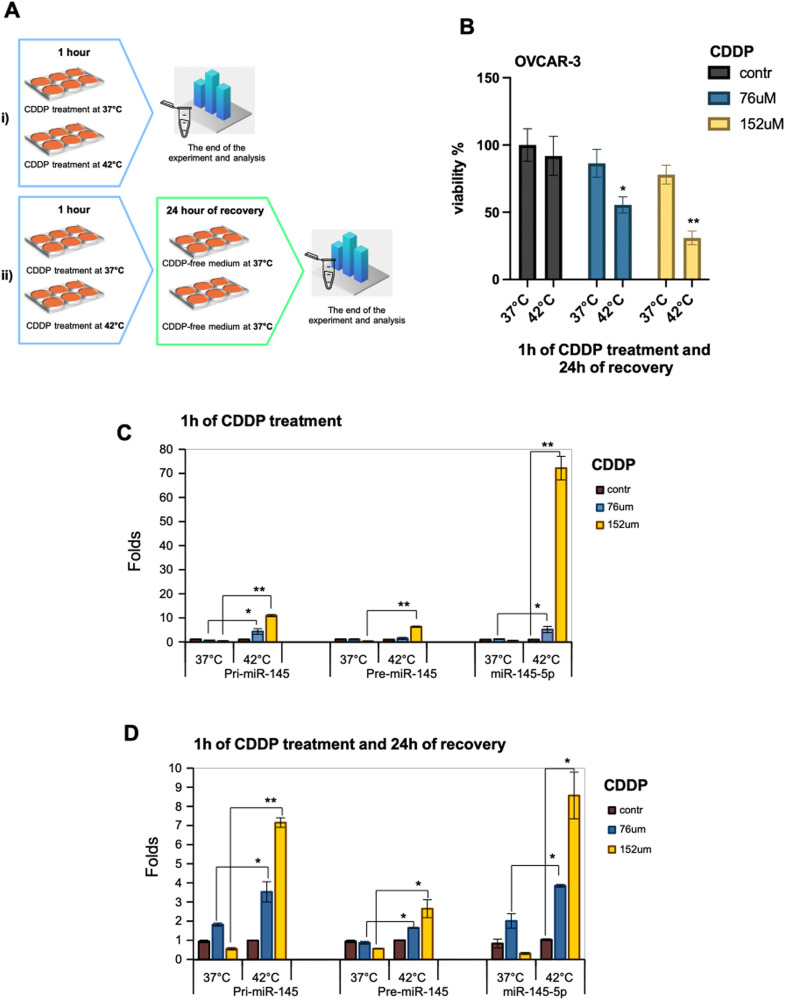
Chemosensitivity of OVCAR-3 cells to HIPEC treatment. **A** HIPEC cisplatin (CDDP) treatment scheme of OVCAR-3 cells. In (i), after 1 h of CDDP treatment at 37 °C or 42 °C, the cells were immediately recovered for the following analysis. In (ii), after 1 h of CDDP treatment at 37 °C or 42 °C, the medium of the cells was replaced with CDDP-free medium, and all the cells were incubated for a recovery time of 24 h at 37 °C. **B** MTT assay in OVCAR-3 cells treated as described in (ii) to test the viability. Values are mean ± SD obtained from 2 separate experiments in quintuplicate. *p* values were calculated with two-tailed *t*-test. Significant *p* values are indicated as *p < 0.05 and **p < 0.01 of 42 °C samples versus 37 °C samples. **C**, **D** Pri-, Pre- and mature miR-145-5p expression was evaluated by RT-qPCR from OVCAR-3 cells treated as described in (i) (**C**) and in (ii) (**D**). *p* values of PCRs were calculated with two-tailed Student’s *t*-test. Statistically significant results are *p < 0.05 and **p < 0.01.

To reproduce the HIPEC treatment in vitro and assess the involvement of miR-145-5p, we planned the following two experimental settings: (i) OVCAR-3 cells were incubated for 1 h at 37 °C and 42 °C in medium containing cisplatin; (ii) OVCAR-3 cells were incubated for 1 h at 37 °C and 42 °C in medium containing cisplatin and after 1 h of incubation the cells were replenished with cisplatin-free medium for 24 h at 37 °C (Fig. 5A). Cisplatin was used at two concentrations as 76 μM and 152 μM, the latter was identified as IC50 for OVCAR-3 cells [35, 36]. We found that OVCAR-3 cells treated as described in the experimental setting (ii) exhibited a significant reduction of cell viability at both cisplatin concentrations when incubated at 42 °C (Fig. 5B). We also assessed the expression of pri-miR-145, pre-miR-145 precursors and mature miR-145-5p in OVCAR-3 within the two above-mentioned experimental settings. As shown in Fig. 5C, both the miR-145 precursors and mature miR145-5p were significantly enriched in OVCAR-3 cells incubated for 1 h at 42 °C with both cisplatin concentrations. A robust accumulation of mature miR-145-5p was evidenced at the cisplatin IC50 treatment (Fig. 5B). In the experimental setting (ii) the accumulation of both pri- and pre-miR-145 precursors and mature miR-145-5p were significantly enriched in cells previously incubated at 42 °C (Fig. 5D).

Altogether, these findings indicated that the production of mature miR-145-5p is a rather early event in such pre-clinical experimental conditions.

### The Heat Shock Transcription Factor-1 (HSF-1) regulates miR-145-5p expression

**Fig. 6:**
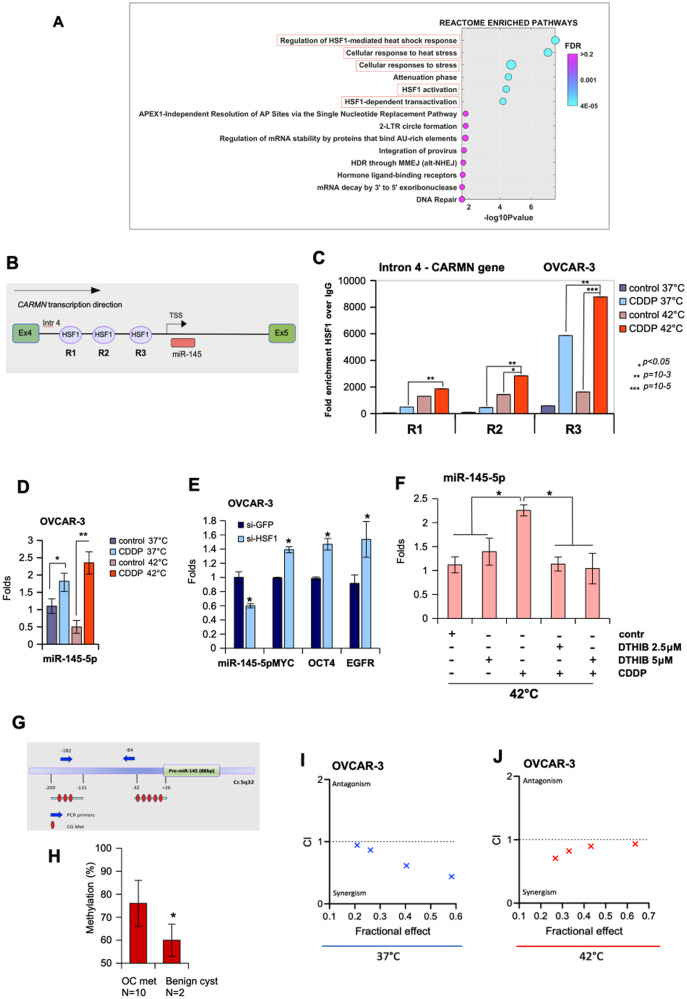
The Heat Shock Transcription Factor-1 (HSF-1) regulates miR-145-5p expression. **A** The REACTOME pathway enrichment analysis of differential expressed genes. 14 statistically significant pathways are listed, and their colors are shown by FDR *q* values. **B** Schematic representation of the intron 4 of the *CARMN* locus gene (Chr. 5_q32; NR_105060) where the 3 consensus sequences evaluated for the recruitment of HSF-1 are indicated as R1, R2, and R3. **C** The cross-linked chromatin purified by OVCAR-3 cells treated as described in Fig. 5A (i) was used in ChIP experiments using anti-HSF-1 antibody and Rabbit IgG as negative control. [CDDP] was 152 μM. A representative experiment was shown. The experiment was performed in biological duplicate. The R1, R2, and R3 region occupancy were analyzed by qPCR. Normalization was performed to the amount of input chromatin. The ChIP samples were further tested by qPCR on a region that was negative for transcriptional factor recruitment. Bars represent mean ± SD from technical triplicates. *p* values were calculated with two‐tailed Student’s t‐test. Statistically significant results are indicated with *p < 0.05. **D** TaqMan assay to evaluate miR-145-5p from the same OVCAR-3 cells used for ChIP experiments. *p* values of the real time PCRs were calculated with two-tailed Student’s *t*-test. Each sample was in technical triplicates and biological duplicates. Statistically significant results are *p < 0.05 and **p < 0.01. **E** TaqMan assay to evaluate the expression of miR-145-5p and its target genes in OVCAR-3 depleted of HSF-1 by si-RNA transfection. Bars represent mean ± SD from biological triplicates. *p* values were calculated with two‐tailed Student’s *t*‐test. Statistically significant results are indicated with *p < 0.05. **F** RT-qPCR to evaluate the expression of miR-145-5p in OVCAR-3 pre-treated with DTHIB (HSF-1 inhibitor) for 72 h and the last 24 h incubated with CDDP. The experiment was conducted at 42 °C. Bars represent mean ± SD from biological triplicates. *p* values were calculated with two‐tailed Student’s *t*‐test. Statistically significant results are indicated with *p < 0.05. **G** Schematic representation of miR-145 promoter regions containing the CpG islands analyzed in 6 F. **H** Histogram showing the average levels of miR-145 regulatory region methylation in peritoneal metastatic lesions (OC met, *n* = 10) and benign cyst samples (Benign cyst, *n* = 2) as control. *p < 0.05 was calculated with two‐tailed Student’s *t*‐test. **I**, **J** Combination index plot of interaction between CDDP and 5-AZA in OVCAR-3 at 37 °C and 42 °C, respectively. The plot was generated by CompuSyn Software.

We have previously shown 46 DEGs after 90 min of HIPEC treatment in patients (Fig. 3H and Supplementary Table 3). Considering this set of genes, REACTOME pathway analysis and Gene Set Enrichment Analysis (GSEA) showed that the most enriched pathways included HSF-1 transcriptional regulation of heat shock response, cellular response to heat stress, HSF-1 activation and related ones (Fig. 6A). TargetMiner analysis highlighted that fifteen (33%) out of 46 differentially expressed gene transcripts could be putative target genes of miR-145-5p (Supplementary Table 3, *genes highlighted in yellow*).

To understand how heat stress mechanistically affected the expression of miR-145-5p, we studied whether HSF-1 transcriptionally regulated miRNA expression. In order, we localized miR-145-5p at the genomic level with the UCSC Genomic Browser. It was found within the intron 4 of the *CARMN* gene (Fig. 6B). We also observed that genomic neighborhood regions of miR-145-5p were characterized by histone H3 acetylation thereby suggesting transcriptional active chromatin (Fig. S3A). This also prompted us to evaluate whether this genomic region could retain transcriptional activity to regulate the expression of miR-145-5p. Using Lasagna search software, we found diverse putative binding sites for HSF-1 in the proximity of putative transcription start site of miR-145-5p (Supplementary Table 4). Interestingly, chromatin immunoprecipitation analyses in OVCAR-3 cells treated with cisplatin for 1 h and incubated either at 37 °C or 42 °C, revealed that HSF-1 was recruited onto the *HSF-1* binding consensus regions (R1, R2, and R3) within the intron 4 of the *CARMN* gene (Fig. 6C). HSF-1 recruitment was much more pronounced when OVCAR-3 cells were treated with cisplatin at 1 h and incubated at 42 °C than at 37 °C, in line with the increased expression of miR-145-5p previously documented under the same conditions (Fig. 6C, D). Interestingly, depletion of HSF-1 expression in OVCAR-3 cells paired with reduced levels of miR-145-5p and the increase of its target genes (Fig. 6E and Supplementary Fig. S3B). No significant modulation of either apoptotic genes or p21 was noted under these conditions (Fig. S3C).

To further consolidate this part of the mechanism, we used DTHIB, a specific HSF-1 inhibitor which binds the DNA binding domain of HSF-1 preventing its recruitment on the target gene promoters, therefore hindering the transcription. In order, we performed a dose-dependence growth curve with OVCAR-3 cells treated at various concentrations of DTHIB at 42 °C, considering that the EC50 of 3 uM was reported for diverse cancer cell lines (Fig. S3D). The DTHIB pretreatment of OVCAR-3 cells prevented the rescue of miR-145-5p expression achieved by CDDP at 42 °C thus far documented (Fig. 6F). This evidence correlated with the fact that under these CDDP/DTHIB co-treatment conditions, the decrease in tumor cell proliferation was no longer observed (Fig. S3E).

We previously reported that reduced miR-145-5p expression in brain metastasis derived from lung, melanoma and ovarian primary tumors was due to methylation of its regulatory regions [22]. Herein, we also evaluated by pyrosequencing assays the methylation status of the CpG island located 200 bp upstream of the regulatory regions of miR-145-5p previously identified (Fig. 6G) in peritoneal metastatic tissues and compared it to that of non-tumoral peritoneal tissues. Pyrosequencing analysis showed a global increase of methylation in all analyzed CpG islands (Fig. 6H). As a control, in the same sequencing assay, we analyzed the methylation of the CpG islands in OVCAR-3 cells treated with vehicle or DNA methyltransferase inhibitor 5 Aza-deoxycytidine (5-AZA, 5uM for 48 h), a DNA demethylating agent. As expected, 5-AZA treatment revealed a change in the methylation status of the CpG islands in the cells (Fig. S3F). These observations may suggest that DNA methylation levels contribute to the low levels of mir-145 in both OC peritoneal carcinomatosis and OVCAR-3 cell line.

### Combined cisplatin and 5-AZA treatments exert synergistic anticancer effects on ovarian cancer cell lines

Promoter hypermethylation was reported to be prognostic of a shorter progression free survival (PFS) and reduced overall survival (OS) in HGSOC patients [37–39]. Preclinical evidence and ongoing clinical trials support the value of epigenetic therapeutic approaches in the treatment of advanced ovarian cancer patients [40].

We aimed to assess whether the rescue of miR-145-5p expression by pharmacologically releasing its promoter methylation could potentiate the anticancer effects of cisplatin. To this end, OVCAR-3 and ES2 cell lines were treated with the 5-AZA that is used as a first-line treatment in many cancers resistant to conventional therapies. We found that 5-AZA treatment increased expression of miR-145-5p and p21 (Supplementary Fig. S4A, B) and led to a reduced cell growth of ovarian cancer cells (Supplementary Fig. S4C, D). Restored expression of miR-145-5p resulted in the concomitant downregulation of its target genes*, EGFR, OCT4, MYC* and *MUC1* (Supplementary Fig. S4B, E). Interestingly, we found that combined treatment with cisplatin and 5-AZA exerted synergistic killing effect on ovarian cancer cells when incubated at 37 °C (Fig. 6I). This synergistic effect was also seen when ovarian cancer cells were incubated at 42 °C mimicking the HIPEC treatment at the preclinical level (Fig. 6J).

Collectively, these preclinical findings might suggest that HIPEC treatment could benefit from the addition of epigenetic drugs such as 5-AZA in the treatment of peritoneal disseminated ovarian cancers.

## Discussion

**Fig. 7:**
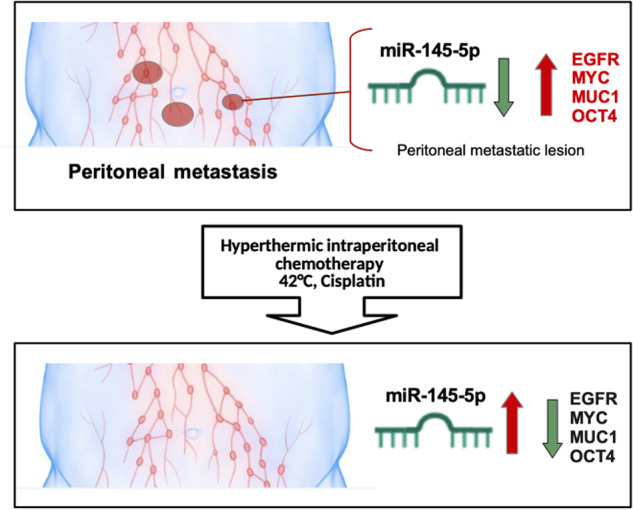
Molecular model. Schematic representation depicting regulation of miR-145-5p and their target genes in peritoneal metastasis upon HIPEC-CDDP treatment.

In the present study, we provide novel insights into the molecular mechanisms through which HIPEC treatment exerted its anticancer effects on ovarian peritoneal carcinomatosis. Indeed, we monitored the expression of miR-145-5p and its target mRNAs, such as c-*MYC, EGFR, MUC1,* and *OCT4* before and during HIPEC treatment of ovarian peritoneal metastatic lesions (Fig. 3A–E). It has previously been reported that miR-145-5p expression is extremely low in HGSO carcinoma tissues and correlates with poor patient prognosis [41, 42]. We found that HIPEC treatment of ovarian peritoneal metastatic lesions restored miR-145-5p expression and concomitantly downregulated that of its target genes. Previous studies reported that the rescue of miR-145 expression reduced EMT and invasion in several kinds of tumor cell lines, including breast cancer, colon cancer, cervical cancer, melanoma, glioblastoma and head and neck carcinoma [21, 43, 44]. The delivery of ectopic miR-145-5p through the development of miRNA-145-based magnetic nanoparticle formulation downregulated oncogenic targets such as *MUC1*, *OCT4*, and *EGFR*, thereby efficiently inhibiting tumor growth and metastases in mice [45, 46]. Our findings revealed that specific tumor suppressor networks, herein exemplified by miR-145-5p and its four target genes, are strongly impaired in ovarian peritoneal metastatic lesions. HIPEC treatment restored the tumor suppression activity of the network comprising miR-145-5p and its target genes *MYC, EGFR, MUC1*, and *OCT4* (Fig. 7).

It has been reported that the mutational landscape of metastasis frequently overlaps with that of the primary tumor, thereby limiting the repertoire of the available targeting drugs to those used for the treatment of the primary tumor. Thus, the study of additional genetic and non-genetic layers of alterations may be pivotal in identifying novel targets to be tackled with precision anticancer drugs. There is growing evidence showing that alterations of the non-coding genome molecules, such as microRNAs, long non-coding RNAs and circular RNAs together with specific pattern of epigenetic modifications might play a central role in the establishment, the maintenance and the chemoresistance of the metastatic phenotype. Our findings, identifying a miR-145-5p/c-MYC/EGFR/MUC1/OCT4 network, provided further evidence demonstrating that the altered activity of non-coding RNAs either as loss of tumor suppressor activity or gain of oncogenic functions might play in the chemoresistance of metastatic lesions.

The value of HIPEC for several types of cancers, including ovarian cancer, is a widely debated topic where its use remains experimental in most national and international guidelines [47]. Some recent meta-analyses showed that the treatment of OC with HIPEC appears to depend on the timing of the last systemic chemotherapy exposure [48]. Hence, while this treatment seems promising there is a need to increase the number of clinical trials for creating application guidelines.

It is likely to expect that the combined treatment of hyperthermia and cisplatin for HIPEC therapy might have a much broader impact on restoring the activation of tumor suppressor pathways in ovarian peritoneal metastatic lesions than that represented by miR-145-5p. Indeed, cancer cells exposed to high temperatures and to other proteotoxic stimuli (e.g., hypoxia, free radicals) activate a “heat shock response” (HS response) whose induction of HS gene expression is mainly transcriptional and is mediated by the heat shock transcription factor HSF-1 [49, 50]. Hyperthermia is known to cause DNA, protein, and membrane damage, to interfere with cell cycle, DNA and protein synthesis, events that may lead to cell death [51]. Indeed, RNA-seq analysis of the metastatic lesions allowed us to identify a selected number of differentially expressed genes that cluster into specific pathways mostly related to heat stress (Figs. 3H and 6A). We identified the transcription factor HSF-1, as a novel transcriptional regulator of miR-145-5p expression, whose heat-induced transcriptional activity might be an important upstream mediator for HIPEC anticancer effects (Fig. 6B, C).

The results highlighted in the pre-clinical experimental conditions (Fig. 5), indicated that the production of mature miR-145-5p after HIPEC is a rather early event. These findings might mimic the rapid induction of mature miR-145-5p that we found in the peritoneal metastatic lesions already after 30 min of in vivo HIPEC treatment. Furthermore, it could indicate that the chain of events leading to the production of mature miR-145-5p through generating pri- and pre-miR precursors is still active in the absence of both heat stress and cisplatin.

Reduced miR-145-5p expression has also been linked to methylation of its regulatory regions [22]. We found that methylation of miR-145-5p was significantly enriched in ovarian peritoneal metastatic tissues when compared to benign peritoneal cysts (Fig. 6H). These findings strongly supported the possibility that the efficacy of HIPEC treatment could benefit from adding methylation inhibitors such as 5-AZA as they could re-activate those tumor suppressor networks impaired by promoter methylation. Interestingly, our preclinical results showing synergistic effects of cisplatin plus 5-AZA on ovarian cancer cells incubated at both 37 °C and 42 °C (Fig. 6I, J) provided further evidence to this possibility. Clinical studies aimed at comparing the efficacy of standard HIPEC, as cisplatin plus hyperthermia versus HIPEC plus 5-AZA on the treatment of ovarian peritoneal metastasis may be worth to fully validate the preclinical findings reported.

## Materials and methods

### Patient samples and HIPEC treatment

The study included 10 cases of ovarian peritoneal carcinomatosis treated with cytoreduction surgery and HIPEC (HIPEC with closed technique, i.e., perfusion of the abdominal cavity with solution containing Cisplatin [CDDP] 75 mg/m^2^ in 2 L/m^2^ of saline heated to 42 °C and pumped at a mean flow of 2000 ml/min for 90 min). In order to be included in the study, clinicians consecutively enrolled patients with metastatic peritoneal nodules showing a high number of blood vessels in the stroma [6]. First, at the moment of the surgery, a small levy of health and tumoral peritoneal tissue had been performed. The two tissues were diagnosed by the pathological anatomy group. At the end of the surgery, the metastasis was removed from the peritoneal cavity, and a single vascularized neoplastic nodule of about 2 × 2 cm was left and was used for the time-course sampling of the HIPEC treatment. The metastatic nodule was lengthwise engraved and divided into six parts for the time course collection. The first biopsy of the neoplastic lesion was performed at time zero (before HIPEC) and the other biopsies were performed during the HIPEC washing of the peritoneal cavity for a total of 5 samples every 15 min (0, 15, 30, 60, and 90 min). The specimens were collected by the surgeon and promptly preserved in RNA-Later (Ambion).

The Ethics Committee of the IRCCS Regina Elena National Cancer Institute approved the study (number: RS56/09). The informed consent forms were obtained from all patients.

Clinical features of the patients and treatments are reported in Supplementary Table 1.

The study was performed in two phases. A flowchart of the study design is illustrated in Fig. 1.

### Immunohistochemistry (IHC)

Paraffin-embedded tissue sections (4 μm in thickness) were subjected to IHC analysis at the Department of Pathology. After carrying out these routine steps, the sections were hybridized with specific antibodies against BRCA1 (6B4, mouse monoclonal antibody, Novus Biological Inc., USA), MYC (9E10, mouse monoclonal, Thermo Scientific, Pittsburgh, PA, USA) and p53 (DO-7, mouse monoclonal, Dako, Agilent Technologies). IHC analysis were performed on Bond II autostainer by using Bond Polymer Refine Detection kit (Leica Biosystems).

### Cell lines and cultures

All cell lines were freshly purchased from the ATCC and tested by PCR for the presence of Mycoplasma. Cells were maintained in culture for no more than six passages. The OVCAR-3 (ATCC HTB-161) (mutp53-R248Q) ovarian cancer cell line was cultured in RPMI 1640 medium (Invitrogen, Carlsbad, CA, USA), 20% (*v/v*) FBS (Invitrogen, Carlsbad, CA, USA) and 10μg/ml Bovin Insulin (Sigma Aldrich); the ES2 (ATCC CRL-1978) (mutp53-S241F) ovarian cancer cell line was cultured in RPMI medium 1640 medium (Invitrogen, Carlsbad, CA, USA) and 10% (*v/v*) FBS (Invitrogen, Carlsbad, CA, USA). All media were supplemented with 100 units/ml penicillin/streptomycin (CliniSciences, Italy). The cells were maintained at 37 °C in a humidified 5% CO_2_ incubator.

### Reagents

To overexpress miR-145-5p in cells, we used *mir*Vana® miRNA mimic (Thermo Scientific, Pittsburgh, PA, USA) (Assay ID: MC11480) and *mir*Vana™ miRNA Mimic, Negative Control #1 (Thermo Scientific, Pittsburgh, PA, USA) (n°4464058).

To inhibit HSF-1 expression, HSF-1 smart pool of three specific siRNA oligos are transduced in ovarian cancer cells (HSF-1 human siRNA sc-35611, Santa Cruz Biotech., Santa Cruz, CA, USA). As a non-silencing control, we transfected si-GFP oligonucleotides (si-GFP target sequence 5′-GGCTACGTCCAggaGCGCACC-3′).

All the cells were transfected by Lipofectamine® RNAiMAX Transfection Reagent following the manufacturer’s instructions.

Cisplatin (CDDP) (n° 232120) and 5-Azacytidine (5-AZA) (2353-33-5) were purchased by Merck KGaA, Darmstadt, Germany (n° A2385) and diluted as specified in the data sheets. HSF-1 inhibitor DTHIB (n° HY-138280) was purchased by MedChemExpress and diluted as specified in the data sheet.

### Cell viability and colony formation assays

Cell viability was carried out by using two methodologies. Cell viability was assayed with ATPlite (Perkin Elmer) according to the manufacturer’s instructions using the EnSpire Multilabel Reader (Perkin Elmer). MTT assay kit was supplied by Abnova (KA1606) following the manufacturer’s instructions. The fluorometry was recorded to 570 nm with a plate reader (Thermo Scientific). Cells (2000 cells per well) were plated in triplicate in 96-well plates and treated as indicated in the corresponding figures.

In the colony-forming assays, cells were transfected as indicated in the figures. After 24 h of transfection, 800 cells from each point were seeded in 12-multiwell plates and grown for 10 days. Cells were stained using crystal violet stain solution (1% crystal violet and 20% methanol) and the colonies evaluated by using ImageJ software (https://imagej.nih.gov/ij/).

### Boyden chamber migration assay

Cells are transfected as indicated in the figures according to the procedures described in the Materials and Methods section. 24 h after transfection, 50,000 cells are suspended in 500 μl serum free medium in trans-wells (PET membrane of 8 μm pore size, Falcon n. 353097) and placed in 24-well plates containing medium with 10% FBS. After 18 h, chambers are washed, and cells are removed from the upper side of the chamber with a cotton swab. Migrated cells are fixed and stained using crystal violet stain solution. Representative images are shown. The average number of migrated cells from 10 representative fields (six replicates per condition) was counted under an optical microscope. *P* values were calculated with two-tailed Student’s *t*-test. Statistically significant results had a *p* < 0.05.

### Cell extracts and western blotting

The procedures used to obtain protein lysates and resolve proteins on acrylamide gels are those as previously described [52]. Western blotting was performed using the following primary antibodies: mouse monoclonal p53 (DO1) (Santa Cruz Biotech.), rabbit monoclonal GAPDH (Santa Cruz Biotech.), mouse monoclonal Actin (Sigma), rabbit monoclonal HSF-1 (Abcam). The immunodetection was performed by using the enhanced chemiluminescence system (ThermoFisher Scientific, Rockford, IL, USA). The acquisition of the chemiluminescence has performed by using Alliance 4.7 by UVITEC (Eppendorf).

Full and uncropped western blots are presented in Supplemental File.

### RNA extraction, cDNA synthesis and RT-qPCR

Total RNAs are isolated from cells and tissues with TRIzol reagent (Invitrogen, USA) and evaluated using NanoDrop 2000 spectrophotometer (Thermo Scientific, Pittsburgh, PA, USA). cDNA synthesis was obtained using SuperScript™ II Reverse Transcriptase kit (Thermo Scientific, Pittsburgh, PA, USA) following the manufacturer’s instructions. RT-qPCR was conducted using SYBR RT-qPCR Master Mix (Applied Biosystems) in a QuantStudio5 and QuantStudio7 Fast Real-Time PCR Systems (Applied Biosystems).

To detect hsa-miR-145-5p, TaqMan™ MicroRNA Assay (Applied Biosystems) was used following the kit assay protocol. RNU48 and RNU6 TaqMan™ Assays were used to normalize the experiments.

The 2^-ΔΔCT^ method for relative quantitation of gene expression was used to determine mRNA expression levels. *β-actin* and *H3 histone* gene expression was used as endogenous controls to standardize mRNA expression. *p* values were calculated with two-tailed Student’s *t*-test from at least three experiments. Statistically significant results are indicated by a *p* < 0.05.

The primer sequences can be found in Supplementary Table 5.

### Chromatin immunoprecipitation assays

ChIP-qPCR analysis was performed as previously described [52, 53]. Briefly, cells were fixed with 1% formaldehyde (Sigma) at room temperature, and chromatin from lysed nuclei was sheared to 600–800 bp fragments using a Bioruptor sonicator (Diagenode). 100 μg of sheared chromatin and 5 μg of antibody plus 40 μl of magnetic beads (Dynabeads® Protein G 10004D Thermo Fisher Scientific) were used for each immunoprecipitated sample. Rabbit monoclonal anti-HSF-1 (Abcam, ab52757) and rabbit anti-IgG (H-270, Santa Cruz sc-66931) are used. Quantitative real-time PCR was carried out with a QuantStudio 5 Fast Real Time PCR Applied Biosystems (Thermo Fisher Scientific) using SYBR qRT-PCR Master Mix (Applied Biosystems). The amount of immunoprecipitated DNA in each sample was calculated as the fraction of the input [amplification efficiency^(Ct INPUT–Ct ChIP)^], and normalized to the IgG control [52, 53]. All reactions were performed in triplicate. *p* values were calculated with two-tailed Student’s *t*-test. Statistically significant results are indicated by a *p* < 0.05.

Primers used for the amplification of the different regulatory regions are listed in Supplementary Table 5.

### RNA and DNA sequencing

The patients chosen for RNA sequencing are highlighted in yellow in Supplementary Table 1. The criterion of choice was determined by the excellent quality of the RNA extracted from the tissues of these patients. The quality of the RNA was assessed with the Bioanalyzer using the Agilent RNA 6000 Nano Kit. Libraries for RNA-Sequencing were prepared employing the TruSeq RNA Exome kit (Illumina) following the manufacturer’s instructions. The quality of the resulting libraries was controlled via the Bioanalyzer (High Sensitivity DNA Kit). The intermediate library before exon enrichment was quantified with the Bioanalyzer using the Agilent DNA 1000 kit, the final library with qPCR. Samples were sequenced in paired-end mode, sequencing from each side 76 bp on a NextSeq 500 instrument.

RNA-seq data were analyzed with “rnaseq” version 1.4.2 pipeline of nf-core community [52] using default parameters. The alignment was performed with Hisat2.

Normalized TPM values were used to determinate gene modulation among different conditions. A Kruskal–Wallis test with post hoc Tukey’s test was applied for comparison among multiple time points and a paired *T*-test was used for comparison between two groups. Principal Component Analysis and unsupervised hierarchical clustering were performed to identify pattern of expressions.

miRNA/mRNA predicted interactions and enrichment analysis were performed by miRWalk version 3 (http://mirwalk.umm.uni-heidelberg.de/search_mirnas/) and enriched pathways were assessed by the Enrichr web tool (https://maayanlab.cloud/Enrichr/).

The QIAamp DNA FFPE Tissue Kit for human tissues was used to extract DNA from FFPE tissues, according to the manufacturer’s protocols. Sequencing aimed to find *TP53* and *BRCA1/2* mutations was performed by using the Ion 540™ Chip Kit (Catalog number: A27765) contains 4 barcoded chips for sample tracking and sequencing with the Ion S5™ XL Sequencing Systems according to the manufacturer’s protocol (Thermo Fisher Scientific). These analyzes were conducted by the company Eurofins Genoma Group (https://www.genomamilano.it/).

### DNA isolation and pyrosequencing analysis

Genomic DNA was purified using the QIAamp DNA FFPE Tissue Kit for human tissues and QIAamp DNA Kit for cells (Qiagen, Hilden, Germany). DNA was quantified with Qubit 4 Fluorometer by Qubit dsDNA HS Assay Kit (ThermoFisher Scientific). Sodium bisulfite modification of 800 ng DNA was obtained using the DNA Methylation kit (Diatech Pharmacogenetics) according to the manufacturer’s protocol. Modified DNA was subjected to PCR amplification of the specific promoter region of hsa-miR-145 using the Corbett Life Science Rotor-Gene™ 6000. The primer sequences are listed in [22]. PCR products were subjected to quantitative pyrosequencing analysis using a PyroMark Q96 ID (Qiagen) according to the manufacturer’s protocol. The pyrosequencing analysis was performed with PyroMarker CpG software 1.0.11 (Qiagen)

### TCGA dataset

Normalized miRNA expression from Agilent arrays of HG-SOC were obtained from the Broad Institute TCGA Genome Data Analysis Center (2016): TCGA data from the Broad GDAC Firehose 2016_01_28 run. Broad Institute of MIT and Harvard. Dataset. 10.7908/C11G0KM9 (http://gdac.broadinstitute.org/runs/stddata__2016_01_28/data/OV/20160128/).

### In silico analysis

To study the genomic localization of miR-145, the UCSC Genome Browser was used (https://genome-euro.ucsc.edu/cgi-bin/hgGateway). LASAGNA Search software (https://biogrid-lasagna.engr.uconn.edu/lasagna_search/index.php) was used for the HSF-1-binding sites in the intron 4 of the *CARMN* gene to genomic sequences. The matrix applied was Transfac. Promoter 2.0 software (https://services.healthtech.dtu.dk/service.php,Promoter-2.0) was used to predict transcription start site of miR-145 into the intron 4.

### Statistics

Each experiment was performed at least three times. Data are presented as the mean ± SD of ≥3 separate experiments. To compare the paired groups, paired *t*-test was used. For in vitro experiments, the differences between groups were analyzed using the Student *t*-test (2-tailed). Statistical analysis and graphs were performed using GraphPad Prism version 9 (GraphPad Software, Inc.). *P* < 0.05 was considered to indicate a statistically significant difference.

## Supplementary information

Supplementary data

Supplementary Table S1

Supplementary Table S2

Supplementary Table S3

Supplementary Table S4

Supplementary Table S5

Reproducibility checklist

## Data Availability

The experimental data sets generated and/or analyzed during the current study are available from the corresponding author upon reasonable request.
