# Supplementary material for: HSF-1/miR-145-5p transcriptional axis enhances hyperthermic intraperitoneal chemotherapy efficacy on peritoneal ovarian carcinosis
Source: Cell Death Dis. 2023 Aug 19;14(8):535. doi: 10.1038/s41419-023-06064-9 (PMC10439938; doi:10.1038/s41419-023-06064-9)
Supplement: Supplementary file 1 — Supplementary data [file 41419_2023_6064_MOESM1_ESM.pdf]

SUPPLEMENTARY DATA

HSF-1/miR-145-5p transcriptional axis enhances hyperthermic intraperitoneal chemotherapy efficacy on peritoneal ovarian carcinosis

Silvia Di Agostino[1], Valeria Canu[2], Sara Donzelli[2], Claudio Pulito[2], Andrea Sacconi[3], Federica Ganci[2], Fabio Valenti[2], Frauke Goeman[4], Stefano Scalera[4], Francesca Rollo[5], Anna Bagnato[6], Maria Grazia Diodoro[5], Enrico Vizza[7], Mariantonia Carosi[5], Beatrice Rufini[2], Orietta Federici[8], Manuel Giofrè[8], Fabio Carboni[8], Paola Muti[9], Gennaro Ciliberto[10], Sabrina Strano[4], Mario Valle[8], and Giovanni Blandino[2]*.

[1]Department of Health Sciences, Magna Græcia University of Catanzaro, 88100 Catanzaro, Italy.
[2]Translational Oncology Research Unit, Department of Research, Diagnosis and Innovative Technologies, IRCCS Regina Elena National Cancer Institute, Rome, Italy.
[3]Clinical Trial Center, Biostatistics and Bioinformatics Unit, IRCCS Regina Elena National Cancer Institute, Rome, Italy.
[4]SAFU Unit, Department of Research, Diagnosis and Innovative Technologies, IRCCS Regina Elena National Cancer Institute, Rome, Italy.
[5]Department of Pathology, IRCCS Regina Elena National Cancer Institute, Rome, Italy.
[6]Preclinical Models and New Therapeutic Agents Unit, IRCCS-Regina Elena National Cancer Institute, Rome, Italy.
[7]Gynecologic Oncology Unit, Department of Experimental Clinical Oncology, IRCCS Regina Elena National Cancer Institute, Rome, Italy.
[8]Department of Digestive Surgery, IRCCS Regina Elena National Cancer Institute, Rome, Italy.
[9]Department of Biomedical, Surgical and Dental Sciences, University of Milan, Milan, Italy.
[10]Scientific Direction, IRCCS Regina Elena National Cancer Institute, 00144 Rome, Italy.

*Corresponding author. Email address: giovanni.blandino@ifo.it (Blandino G.)

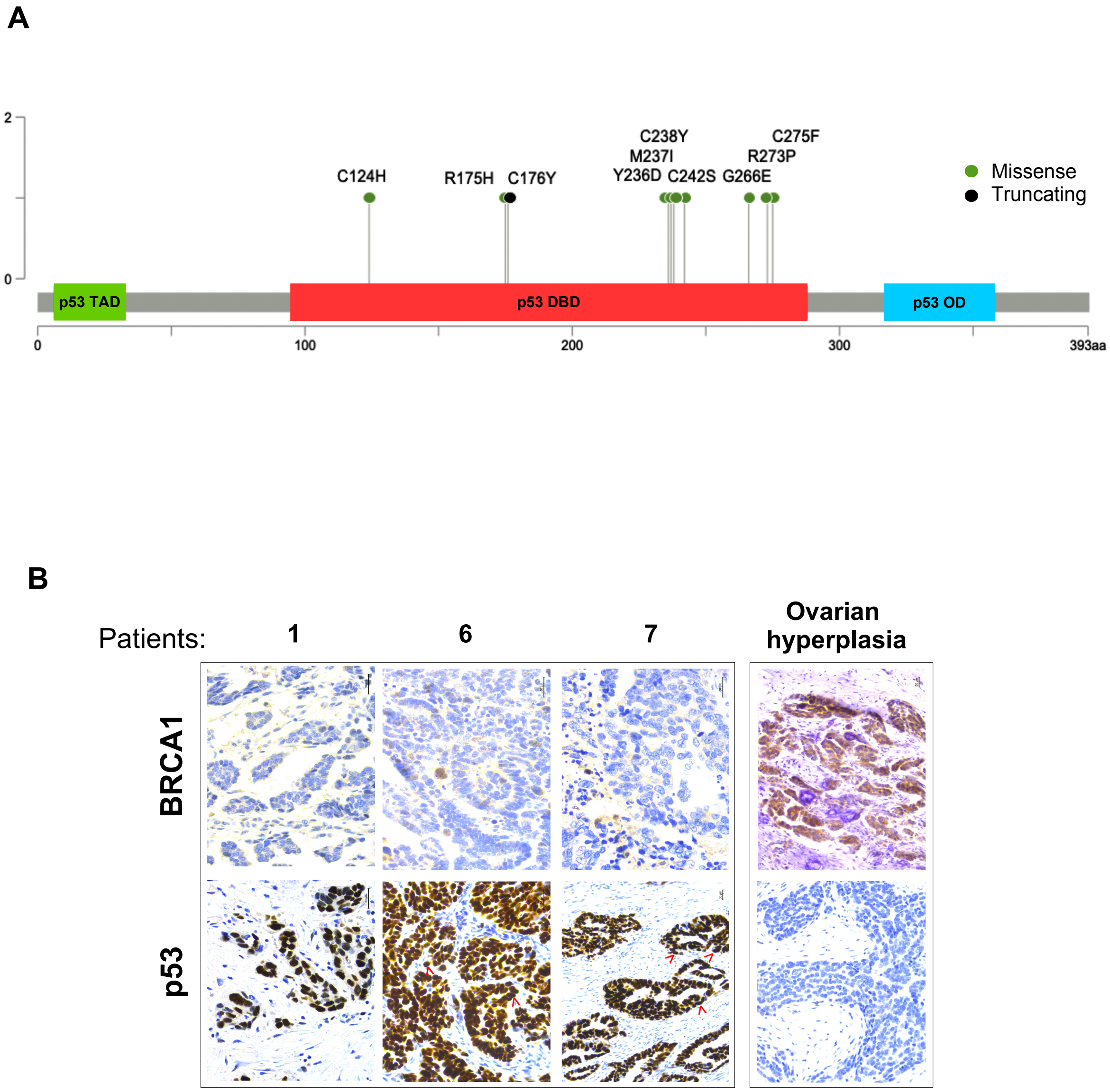

Suppl. Figure S1:
(A) Lollipop plot illustrating *TP53* mutations in ovarian peritoneal metastasis from IRE patients (n=10). Protein domains of p53: TAD, trans-activation domain; DBD, DNA binding domain; OD, oligomerization domain. (B) Staining by H&E and IHC for the indicated proteins of peritoneal metastasis from representative tissues of IRE patients. Tissue from ovarian dysplasia was hybridized with the same antibodies as positive control. Original magnification, 40x. Red arrows indicate examples of cells with complete nuclear staining of p53.

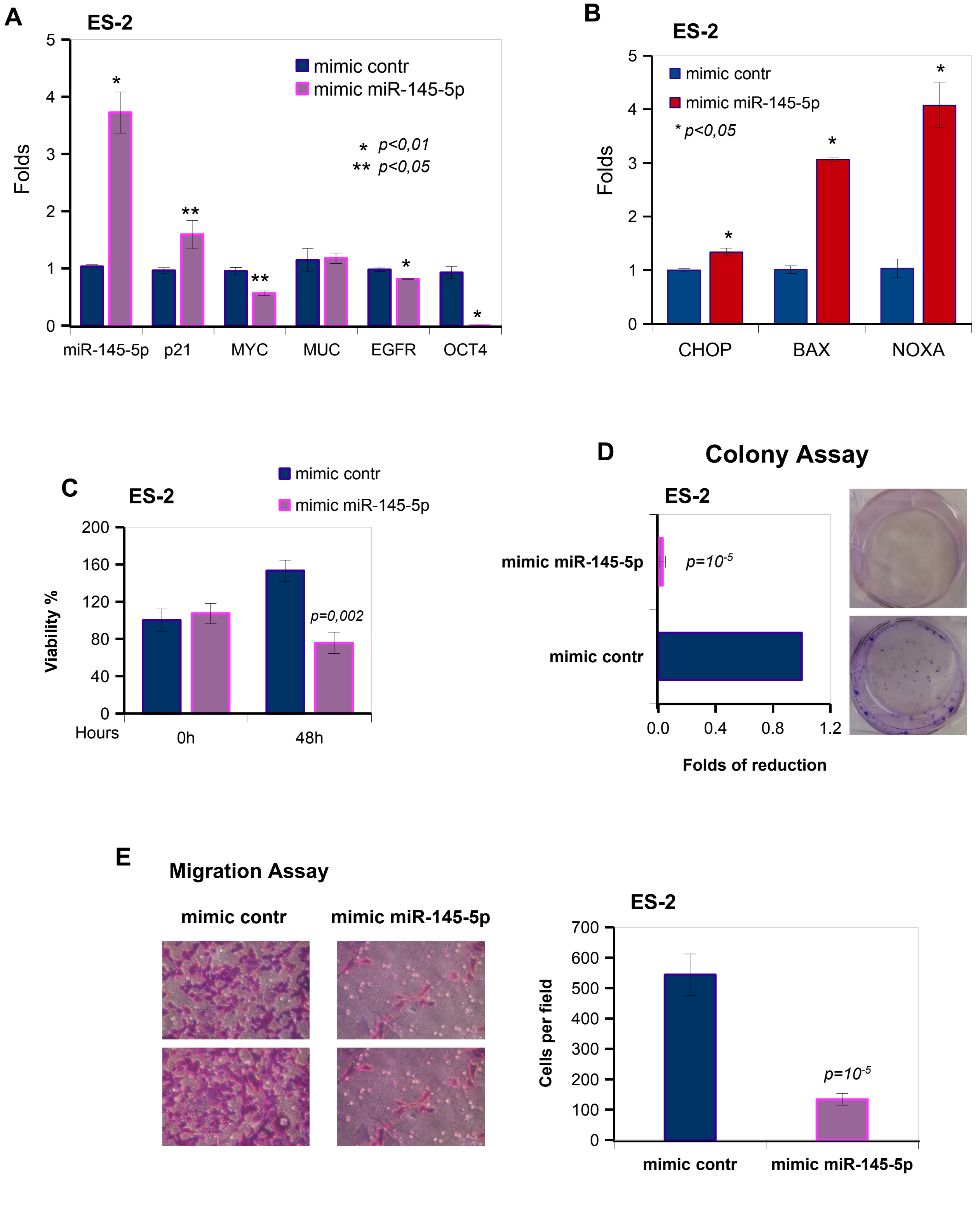

Suppl. Figure S2:

(A,B) ES-2 cell line was transfected with oligonucleotides overexpressing miR-145-5p (mimic miR-145-5p) and mimic control as negative sample. miR-145-5p, MYC, MUC1, OCT4, EGFR and p21 expression are analyzed by RT-qPCR in (A) and CHOP, BAX and NOXA gene expression are showed in (B). p-values were calculated with two-tailed Student's t-test. Statistically significant results are indicated in the figures. (C) Viability of ES-2 cells transfected with mimic miR-145-5p or mimic control was tested by MTT assay at the indicate time points. Values are mean ± SD obtained from 2 separate experiments in quintuplicate. p-values were calculated with two-tailed t-test. (D) Colony formation assay was performed in ES-2 cells transfected with mimic control and mimic-miR-145-5p. The data was obtained by analysing the colonies with ImageJ software. p-values were calculated with two-tailed t-test. (E) Representative images and quantification of the transwell migration assay by Boyden chamber in ES-2 cells. Magnification, 40x. Data in the graph represent the mean ± SD from three biological replicates of the transwell migration assay, each point repeated in technical quadruplicates. p-values were calculated with two-tailed t-test.

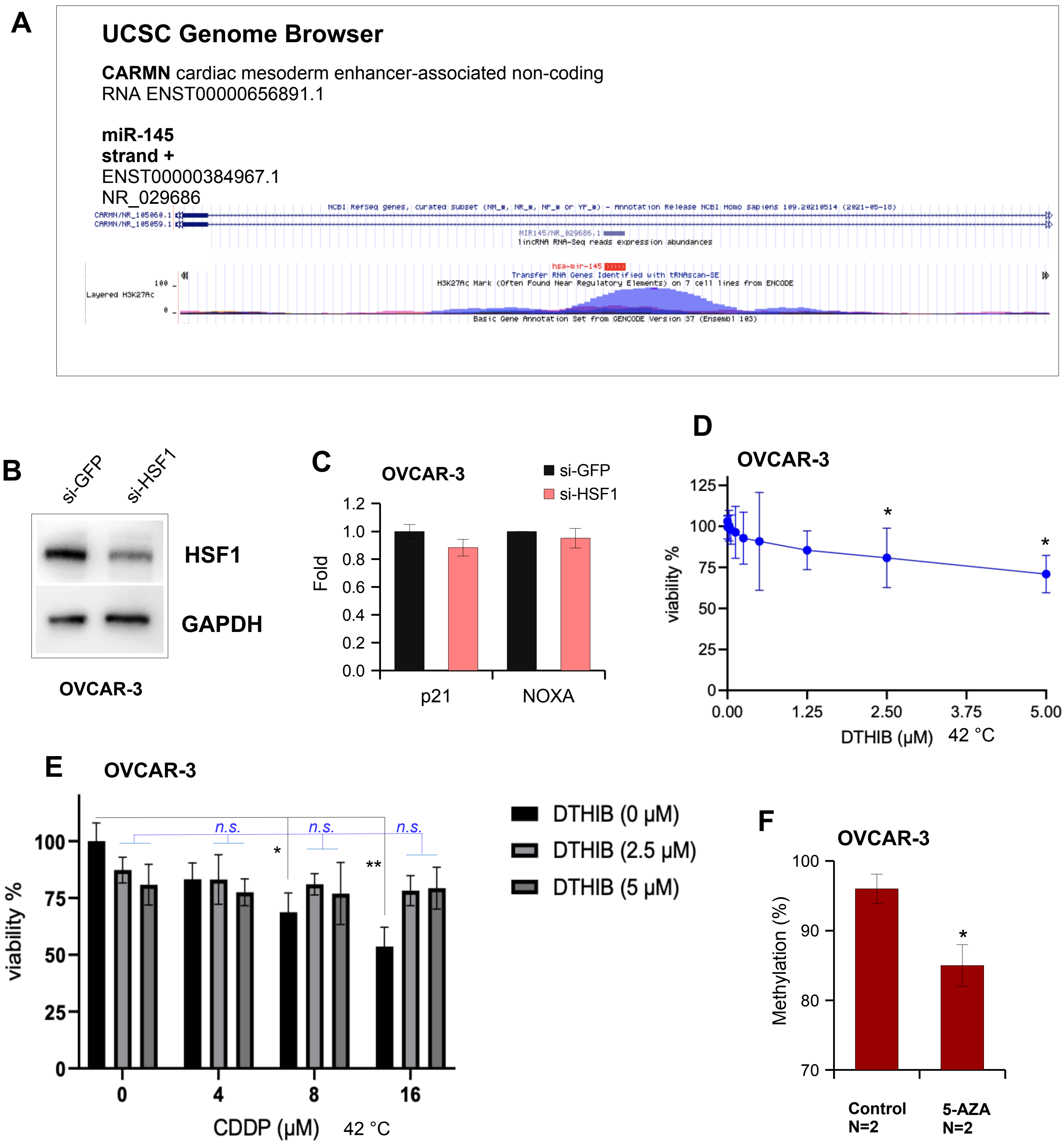

Suppl. Figure S3:
(A) Genomic region from UCSC genome browser of the third intron in the *CARMN* (chr5:149,406,688-149,432,949; GRCh38/hg38), the miR-145 host gene. In red the exact position of miR-145 sequence and the ChIP-seq data profile of H3K27Ac in the third intron. (B) Immunoblotting of HSF-1 and GAPDH (loading control) proteins from whole protein lysates of OVCAR-3 cells transfected with si-GFP and si-HSF-1 smart pool oligos for 48 hours. A representative image is shown. (C) p21 and NOXA expression are analyzed by RT-qPCR in OVCAR-3 where HSF1 expression was downregulated. p-values were calculated with two-tailed Student's t-test from biological triplicates. The differences are not statistically significant. (D) Label-free assay (ATPlite) in OVCAR-3 cells treated for 72h with increasing amount of DTHIB. ATP amount was expressed as percentage of viability. Values are mean ± SD obtained from three separate experiments in quintuplicate. p-values were calculated with two-tailed t-test. Significant p-values are indicated as *p-value < 0.05 versus the control sample without treatment. (E) ATPlite viability assay of OVCAR-3 cells pre-treated with DTHIB (HSF-1 inhibitor) for 72h and the last 24h with CDDP. The experiment was conducted at 42°C. Values are mean ± SD obtained from 2 separate experiments in quintuplicate. p-values were calculated with two-tailed t-test. * $p < 0.05$; ** $p < 0.01$; n.s. was ot significant. (F) miR-145 promoter methylation levels in the OVCAR-3 cell line before (Control) and after treatment with 5 μM of 5-AZA for 48 h. Error bars indicate the standard deviation of two different experiments. * $p < 0.05$.

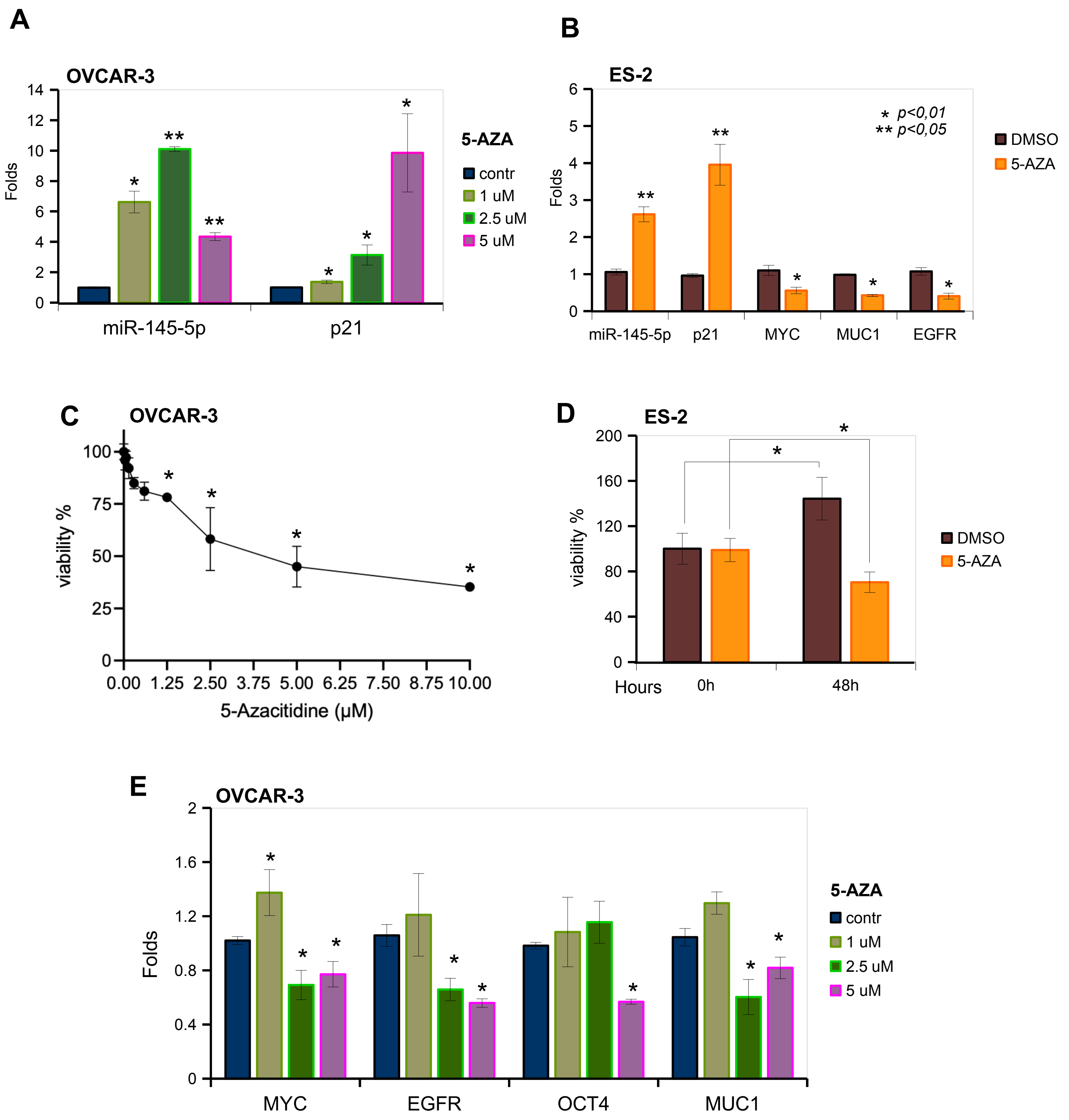

Suppl. Figure S4:
miR-145-5p and p21 expression were analyzed by RT-qPCR in cDNA derived from OVCAR-3 cells treated for 48 hours with 5-AZA at the indicated concentrations. p-values were calculated with two-tailed Student's t-test. Statistically significant results are *p<0.05 and **p<0.01. (B) ES-2 cells were trated for 48h with 5 μM of 5-AZA. RT-qPCR was carried out for the indicated genes. p-values were calculated with two-tailed Student's t-test. Statistically significant results are *p<0.05 and **p<0.01. (C) Label-free assay (ATPlite) in OVCAR-3 cells treated with increasing amount of 5-AZA. ATP amount was expressed as percentage of viability. Values are mean ± SD obtained from three separate experiments in quintuplicate. p-values were calculated with two-tailed t-test. Significant p-values are indicated as *p-value < 0.01 versus the control sample without treatment. (D) ATPlite in ES-2 cells treated for 48h with 5 μM of 5-AZA. p-values were calculated with two-tailed Student's t-test. Statistically significant results are *p<0.05. (E) MYC, EGFR, OCT4, and MUC1 expression were analyzed by RT-qPCR in cDNA derived from OVCAR-3 cells treated for 48 hours with 5-AZA at the indicated amounts. p- were calculated with two-tailed Student's t-test. Statistically significant results are *p<0.05.

Original western blotting data

Fig. S3B

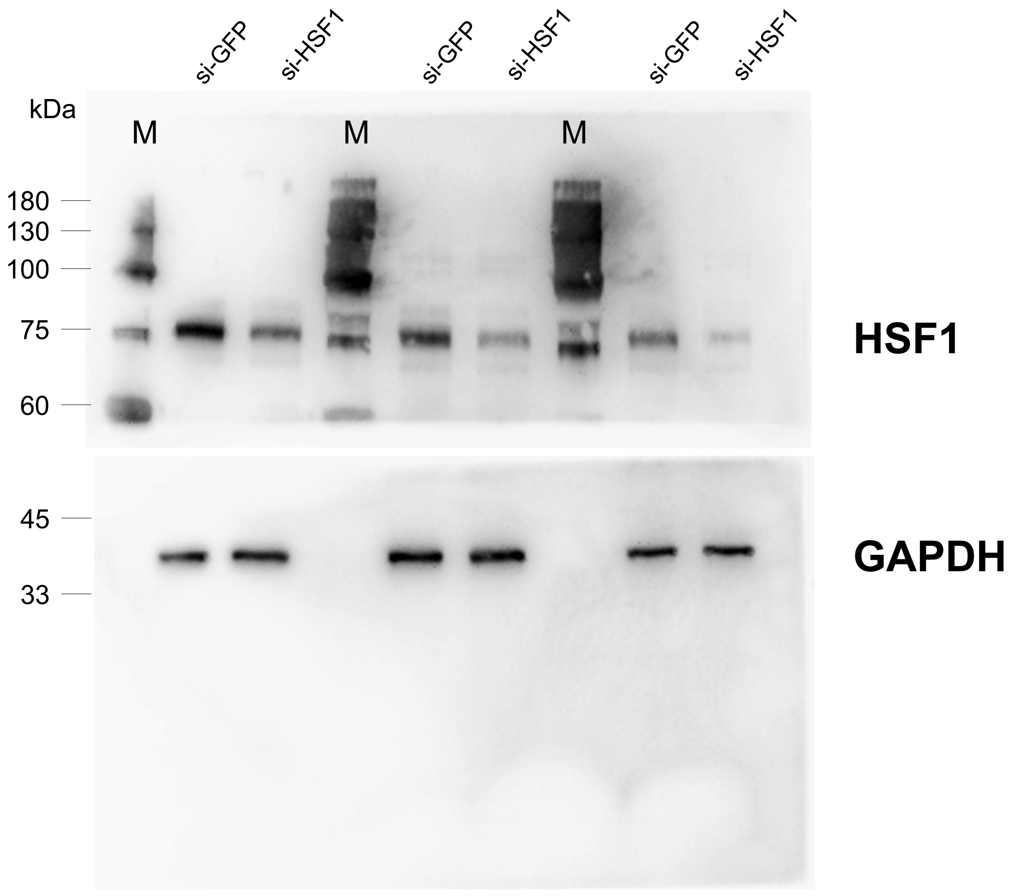

Original immunoblotting of HSF-1 and GAPDH from whole protein lysates of OVCAR-3 cells in biological triplicate.
M: Protein ladder Mid-range molecular weight (10 - 180 kDa) (Abcam ab116027)
