# Supplementary material for: HSF-1/miR-145-5p transcriptional axis enhances hyperthermic intraperitoneal chemotherapy efficacy on peritoneal ovarian carcinosis
Source: Cell Death Dis. 2023 Aug 19;14(8):535. doi: 10.1038/s41419-023-06064-9 (PMC10439938; doi:10.1038/s41419-023-06064-9)
Supplement: Supplementary file 3 — Supplementary Table S2 [file 41419_2023_6064_MOESM3_ESM.docx]

| **Sample** | **Locus** | **Genes** | **Coding** | **Genes** | **Amino Acid Change** |
| --- | --- | --- | --- | --- | --- |
|  |  |  |  |  |  |
| **1** | chr17:7578406 | TP53 | c.524G>A | TP53 | p.Arg175His |
|  |  |  |  |  |  |
| **2** | chr13:32954209 | BRCA2 | c.9183A>T | BRCA2 | p.Leu3061Phe |
|  | chr17:7578397 | TP53 | c.527G>A | TP53 | p.Cys176Tyr |
|  |  |  |  |  |  |
| **3** | chr17:7577568 | TP53 | c.713G>A | TP53 | p.Cys238Tyr |
|  |  |  |  |  |  |
| **4** | chr17:7577575 | TP53 | c.706T>A | TP53 | p.Tyr236Asn |
|  | chr17:41244826 | BRCA1 | c.2722G>T | BRCA1 | p.Glu908Ter |
|  |  |  |  |  |  |
| **5** | chr17:41251790 | BRCA1 | c.547+2T>A | BRCA1 |  |
|  |  |  |  |  |  |
| **6** | chr17:7577120 | TP53 | c.818G>C | TP53 | p.Arg273Pro |
|  |  |  |  |  |  |
| **7** | chr17:7577556 | TP53 | c.725G>C | TP53 | p.Cys242Ser |
|  |  |  |  |  |  |
| **8** | chr17:7577570 | TP53 | c.711G>T | TP53 | p.Met237Ile |
|  | chr17:7579316 | TP53 | c.369_370delTT | TP53 | p.Cys124HisfsTer24 |
|  | chr17:41276049 | BRCA1 | c.65T>C | BRCA1 | p.Leu22Ser |
|  |  |  |  |  |  |
| **9** | chr17:7577141 | TP53 | c.797G>A | TP53 | p.Gly266Glu |
|  |  |  |  |  |  |
| **10** | chr17:7577114 | TP53 | c.824G>T | TP53 | p.Cys275Phe |
|  | chr17:41243029 | BRCA1 | c.4117G>T | BRCA1 | p.Glu1373Ter |
| **11 (Hyperplasia)** | NEGATIVE |  |  |  |  |

**Supplemental Table 2:** Results of *TP53* and *BRCA1/2* gene sequencing in the cohort of metastatic tissues
